# Investigating cortico‐subcortical circuits during auditory sensory attenuation: A combined magnetoencephalographic and dynamic causal modeling study

**DOI:** 10.1002/hbm.25134

**Published:** 2020-07-14

**Authors:** Lingling Hua, Marc Recasens, Tineke Grent‐'T‐Jong, Rick A. Adams, Joachim Gross, Peter J. Uhlhaas

**Affiliations:** ^1^ Institute for Neuroscience and Psychology University of Glasgow Glasgow UK; ^2^ Department of Child and Adolescent Psychiatry Charité‐Universitätsmedizin Berlin Berlin Germany; ^3^ Centre for Medical Image Computing, Department of Computer Science University College London London UK; ^4^ Institute of Biomagnetism and Biosignal analysis Westphalian Wilhelms University Muenster Münster Germany

**Keywords:** dynamic causal modeling, M100, magnetoencephalography, movement‐related magnetic fields (MRMFs), sensory attenuation

## Abstract

Sensory attenuation refers to the decreased intensity of a sensory percept when a sensation is self‐generated compared with when it is externally triggered. However, the underlying brain regions and network interactions that give rise to this phenomenon remain to be determined. To address this issue, we recorded magnetoencephalographic (MEG) data from 35 healthy controls during an auditory task in which pure tones were either elicited through a button press or passively presented. We analyzed the auditory M100 at sensor‐ and source‐level and identified movement‐related magnetic fields (MRMFs). Regression analyses were used to further identify brain regions that contributed significantly to sensory attenuation, followed by a dynamic causal modeling (DCM) approach to explore network interactions between generators. Attenuation of the M100 was pronounced in right Heschl's gyrus (HES), superior temporal cortex (ST), thalamus, rolandic operculum (ROL), precuneus and inferior parietal cortex (IPL). Regression analyses showed that right postcentral gyrus (PoCG) and left precentral gyrus (PreCG) predicted M100 sensory attenuation. In addition, DCM results indicated that auditory sensory attenuation involved bi‐directional information flow between thalamus, IPL, and auditory cortex. In summary, our data show that sensory attenuation is mediated by bottom‐up and top‐down information flow in a thalamocortical network, providing support for the role of predictive processing in sensory‐motor system.

## Graphical abstract

We examined auditory sensory attenuation using a combination of magnetoencephalography (MEG) and computational modeling. Our data show that sensory attenuation is mediated by bottom‐up and top‐down information flow in a thalamocortical network, providing support for the role of predictive processing in sensorimotor systems.

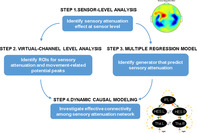

## 1 INTRODUCTION

An important goal of organisms is to distinguish between sensory information originating from the external environment versus sensations caused by the organism's own actions (Schafer & Marcus, 1973; von Holst & Mittelstaedt, 1950). One example to illustrate this phenomenon is sensory attenuation whereby sensations that are self‐generated are decreased in intensity compared with externally‐generated stimuli (von Holst & Mittelstaedt, 1950).

The first framework to account for sensory attenuation was proposed by Von Holst and Mittelstaedt (1950) who suggested that an efference copy of the motor command is used to predict the forthcoming sensory outcome, followed by a comparison with the afferent information (corollary discharge) (Sperry, 1950). From this perspective, sensory attenuation occurs if the predicted sensory feedback matches the incoming sensory stimulus. More recent accounts have highlighted the role of hierarchical inferences in sensory attenuation from a predictive coding perspective (Friston & Kiebel, 2009; Rao & Ballard, 1999).

Sensory attenuation has been observed in tactile, auditory, and visual domains in a range of species (Blakemore, Wolpert, & Frith, 1998; Crapse & Sommer, 2008; Hughes & Waszak, 2011; Poulet & Hedwig, 2002; Schneider, Nelson, & Mooney, 2014), including humans (Blakemore, Wolpert, & Frith, 2000; Limanowski, Sarasso, & Blankenburg, 2018; Synofzik, Lindner, & Thier, 2008), suggesting an evolutionary conserved mechanism. In electro/magnetoencephalographical (EEG/MEG) recordings, auditory sensory attenuation is characterized by the suppression of the N/M100 event‐related potential/field (ERP/ERF) during self‐generated speech or tones (Cao, Thut, & Gross, 2017; Heinks‐Maldonado, Nagarajan, & Houde, 2006; Martikainen, Kaneko, & Hari, 2004). Analysis of the underlying generators identified the superior temporal cortex (ST) as the primary region contributing to the attenuation of the M100 (Aliu, Houde, & Nagarajan, 2009; Martikainen et al., 2004). Moreover, impaired sensory attenuation has been linked to psychiatric disorders, such as schizophrenia (ScZ) (Ford et al., 2001; Ford, Gray, Faustman, Roach, & Mathalon, 2007; Whitford et al., 2017), to account for disturbances in the sense of agency that could potentially underlie the emergence of hallucinations and delusions (Ford & Mathalon, 2005).

von Holst and Mittelstaedt (1950) proposed that motor areas generate an efference copy that is compared with the incoming sensory signal. This is supported by studies with transcranial magnetic stimulation (TMS) showing that interference with motor regions is associated with reduced sensory attenuation in auditory cortex (Haggard & Whitford, 2004). It is currently unclear, however, at which stage motor information impacts sensory processing as this could occur before motor execution (Schneider et al., 2014; Timm, SanMiguel, Keil, Schröger, & Schönwiesner, 2014), during motor action (Stenner, Bauer, Heinze, Haggard, & Dolan, 2015), or following the re‐afference stage of motor action (Burin et al., 2017; Kilteni & Ehrsson, 2017a, 2017b).

In addition to auditory and motor areas, the parietal cortex (Pollok, Gross, Kamp, & Schnitzler, 2008) as well as subcortical areas, such as the thalamus (Sherman, 2016) and cerebellum (Cao, Veniero, Thut, & Gross, 2017), have been involved in sensory attenuation. There is evidence to suggest that the inferior parietal cortex together with the cerebellum predicts the sensory outcomes of actions (Blakemore & Sirigu, 2003; Pollok et al., 2008). The thalamus, on the other hand, has been postulated to be involved in the relay of the efference copy generated in motor areas to auditory regions (Sherman, 2016). This hypothesis is supported by findings from visual perception where lesions in the thalamus lead to impaired saccade orientation, possibly through interfering with updating the corollary discharge signal (Sommer & Wurtz, 2004).

In the current study, we aimed to provide novel insights into the contributions of cortical and subcortical regions as well as their interactions toward auditory sensory attenuation through the combination of advanced source reconstruction of MEG data together with computational modeling. To address these questions, we first compared M100 responses during self versus non self‐generated 40 Hz amplitude modulated (AM) tones. We then identified movement‐related magnetic fields (MRMFs) in order to identify potential efferent motor signal contributions to sensory attenuation. MRMFs have not been investigated within this paradigm, but can be identified and extracted from MEG data (Nagamine et al., 1994). Multiple regression analyses were used to identify the contribution of motor cortical regions towards the attenuation of the M100 amplitude in auditory areas. Finally, we employed dynamic causal modeling (DCM) (Friston Harrison & Penny, 2003) to study the interactions between sources underlying sensory‐attenuation in MEG‐data.

Based on existing evidence and theoretical models, we predicted that, in addition to auditory cortex, sensory attenuation would engage a distributed cortical‐subcortical network. Moreover, we anticipated that this network would involve both bottom‐up as well as top‐down mediated interactions, providing support for the role of predictive processes in sensory attenuation.

## 2 METHODS

### 2.1 Participants

Thirty‐five healthy volunteers were recruited from the University of Glasgow and provided informed consent prior to the experiment. All participants were right‐handed (26 females/9 males; mean age: 22.3 years) and were characterized by normal hearing levels and without a history of psychiatric disorders. Handedness was assessed with Edinburgh Handedness Inventory (Oldfield, 1971).

### 2.2 Experimental paradigm

**FIGURE 1:**
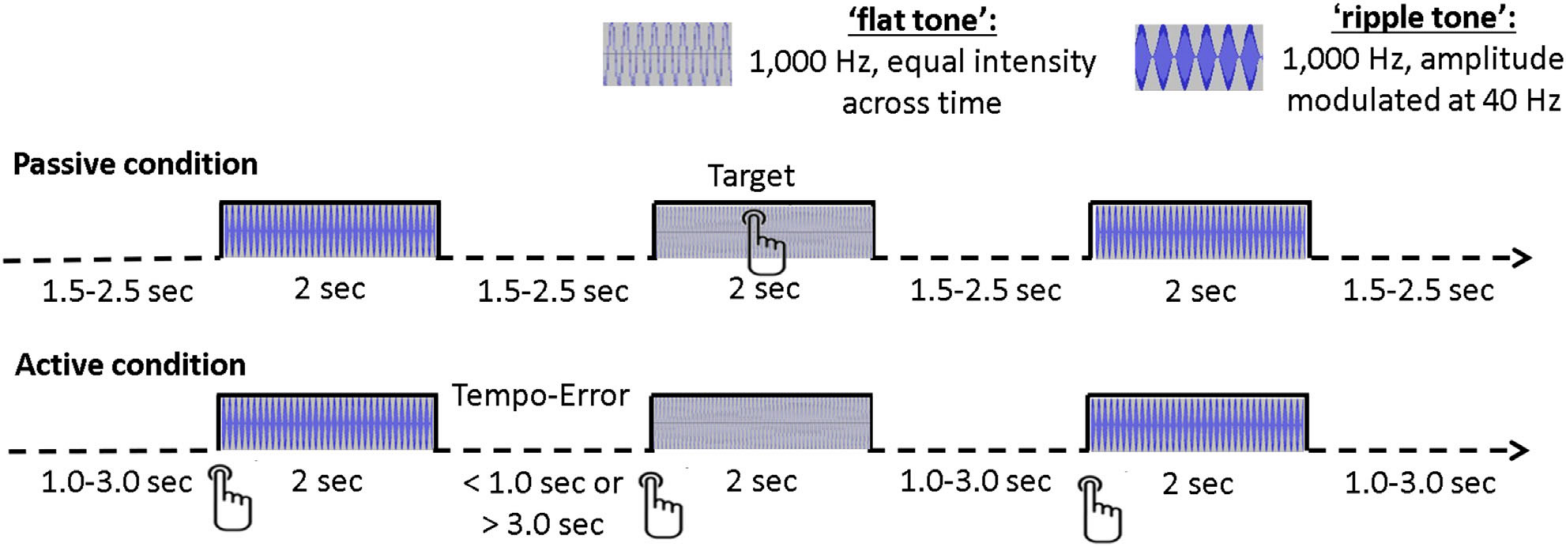
Experimental paradigm. In the passive condition, participants responded to 10 “flat” tones (1,000 Hz, 2,000 ms duration, 93 dB) and passively listened to 100 “ripple” tones (40 Hz amplitude‐modulated 1,000 Hz carrier tones, 2,000 ms duration, 87 dB) and with an average jittered stimulus‐onset‐asynchrony (SOA) of 4,000 ms (3,500–4,500 ms). In the active condition, 100 ripple tones were elicited through a button press at ~4,000 ms SOA (between 3,000 and 5,000 ms). A flat tone was presented in this condition when the response was outside the SOA

A 1,000 Hz, flat tone of constant intensity (2000 ms duration, 93, dB) and a 40 Hz amplitude‐modulated; 1,000 Hz tone (“ripple” tone, 2000 ms duration, 87 dB) were presented binaurally in two blocks with 100 trials each: 1) A “passive” condition block compromising of 100 ripple tones and 10 flat tones with a jittered stimulus‐onset‐asynchrony (SOA) between 3,500 and 4,500 ms. Participants were instructed to press a button with their right index finger when a flat tone occurred and 2) A self‐generated condition (“active” condition) that required participants to elicit a ripple tone via button press with their right index finger at approximate 4,000 ms SOA. A flat tone was presented if the participant responded earlier than 3,000 ms or later than 5,000 ms SOA (Figure 1). Prior to the beginning of the experiment, participants were given practice runs to familiarize themselves with the task.

### 2.3 Data collection and analysis

MEG‐data were acquired with a 248‐magnetometers whole‐head MEG system (MAGNES 3,600 WH, 4‐D Neuroimaging) at a sample rate of 1,017.25 Hz, and filtered online between direct current (DC) and 400 Hz. Prior to the MEG‐recording, the head‐shape and five head position indicator (HPI) coils was digitized using a Polhemus Fastrack digitizer. Head position was recorded at the beginning and the end of each block.

A 3D MPRAGE sequence were used to collect the T1‐weighted structural magnetic resonance imaging (sMRI) data on a 3 Tesla scanner (Siemens, Tim Trio System). The parameters were: 1 × 1 × 1 mm resolution, 192 volumes, TR = 2,250 ms, TE = 2.6 ms, FA = 9°.

### 2.4 Sensor‐level analysis

All analysis were conducted with the Fieldtrip‐toolbox (fieldtrip‐20170110) (Oostenveld, Fries, Maris, & Schoffelen, 2011). Only trials that contained a ripple tone were included in sensor and source‐space analysis. For the preprocessing of MEG‐data, recordings were filtered to remove line noise at 50, 100, 150 Hz using a discrete Fourier transform filter, and were epoched from −1,000 to 3,000 ms. Trials with artifacts were detected manually and rejected from further analysis. Faulty sensors with large signal variance or whose signals were flat were removed and interpolated using the nearest‐neighbor averaging procedure.

Independent component analysis (ICA) was applied to remove variance due to artifacts such as heartbeat, saccade and eye blinks (Rejected ICA components in the passive condition: mean/median ± *SD* = 6.0/5.0 ± 3.4, range [2 15]; active condition: 5.3/5.0 ± 1.9, [3 11]). Prior to trial averaging (trials removed: passive condition: 2.8/3.0 ± 2.8, [0 11]; active condition: 4.0/5.0 ± 3.2, [0 18], MEG‐data were band‐pass filtered with a butterworth filter (1–30 Hz, filter direction “two‐pass,” filter order 6), and then baseline corrected from −700 to −200 ms, followed by averaging of individual trials in each condition. Filtered neuromagnetic data were transformed from axial magnetometer to planar gradient signals (Bastiaansen & Knösche, 2000).

### 2.5 Source‐space analysis

Individual T1‐weighted MRI data were firstly manually aligned with MEG axial‐data with three anatomical landmarks (the nasion, right and left ears preauricular points), followed by an automatic co‐registration procedure with the ICP algorithm (Besl & McKay, 1992). A single‐shell volume conductor model was utilized for individual head models. The head model was further warped into a three‐dimensional template grid (6 mm resolution grid) in Montreal neurological institute (MNI) coordinates to normalize the source position and reduce individual differences (Nolte, 2003).

Source‐space (virtual channel) data were extracted based upon the centroids of 116 available AAL atlas regions from BrainNet Viewer software (Xia, Wang, & He, 2013), followed by warping into individual normalized MRI to extract signals at each brain region. The linear constraint minimum variance (LCMV) beamformer was used to compute the source‐space data with the covariance matrix based on the time window from −1,000 to 3,000 ms. The regularization value of the covariance matrix was set to 5%. Finally, Singular value decomposition (SVD) was used to decompose and extract the data vector representing the dominant source orientation.

Source‐space data were band‐pass filtered (butterworth) from 1 to 30 Hz in a two‐pass direction with a steepness order of 6 (default). Subsequently, the filtered data were baseline corrected from −700 to −200 ms before averaging across trials. To identify the analysis window for differences between active and passive conditions (sensory attenuation), we used a cluster‐based nonparametric permutation approach to detect the best‐fitting window across auditory regions between of 50 and 200 ms.

### 2.6 MRMFs

To identify the motor areas involved in sensory attenuation, we averaged the source‐space data across trials and participants in order to identify MRMFs (Jankelowitz & Colebatch, 2002; Nagamine et al., 1994). MRMFs were visually examined across all virtual channels and MRMFs were identified according to their peak latency. We selected four MRMFs with the largest amplitude in five regions of interest (ROIs) that were entered into a regression analysis to examine the relationship with sensory attenuation of the M100 in auditory regions. Movement‐related cortical areas were not used as DCM requires the driving input to be the same between experimental conditions (see below).

### 2.7 DCM analysis

DCM was used to explore the causal interactions between brain regions that explain differences between observed ERFs (David et al., 2006). Conceptually, the interactions between neural nodes in DCM consist of (a) Structural forward, backward and lateral connections between nodes which convey changes in brain activity elicited by a stimulus (i.e., a driving input) and (b) Modulatory connections which estimate the effect of experimental factors (context‐dependent) on neural interactions, including forward, and backward connections to investigate the contribution of bottom‐up message passing versus top‐down mediated predictions towards sensory attenuation. In addition, self‐modulation within each source was added to test the role of intrinsic changes in neural excitability (Kiebel, Garrido, & Friston, 2007) as well as the contribution of lateral connections given their role in auditory processing (Boly et al., 2011; Phillips, Blenkmann, Hughes, Bekinschtein, & Rowe, 2015).

DCM‐analysis of evoked responses uses excitatory and inhibitory neuronal subpopulations in a neural mass model was applied to auditory ERF responses between −100 and 200 ms. Source‐space data were entered into the DCM analysis, which was performed under the “LFP” spatial model setting (used to model relationships in either real or virtual electrode data). Given that we were interested in the changes in connection strengths during sensory attenuation relative to a baseline condition (auditory input without sensory attenuation), between‐condition effects were set to 0 (baseline) and 1. DCM was performed based on Statistical Parametric Mapping 12 (SPM 12,v7487) (https://www.fil.ion.ucl.ac.uk/spm/).

### 2.8 Statistics

#### 2.8.1 Sensory attenuation effect

Sensor‐level sensory attenuation effects were examined with a cluster‐based nonparametric *t* test implemented in Fieldtrip (Maris & Oostenveld, 2007). We focused on a time window between 110 and 140 ms, which was identified based on visual inspection of the grand‐average data and covered the peak latency of the M100 component. Significant clusters were calculated with the Monte Carlo method with 1,000 permutations (*p* < .05, alpha‐level = 0.05, two‐tailed).

Due to the fact that the latency of the M100 at source level was slightly different across auditory regions, we used a cluster‐based nonparametric permutation approach to detect significant difference between active and passive conditions between 50 and 200 ms. A false discovery rate (FDR) was applied to correct for multiple comparisons across 116 source regions (*p* < .05, alpha‐level = 0.05, two‐tailed).

#### 2.8.2 Regression analysis

A stepwise multiple regression method was employed to identify the relationship between MRMFs and attenuation of the M100 amplitude. The dependent variables were M100 sensory attenuation in right HES and right ST which was calculated through the root mean square (RMS) of M100 amplitude. Due to the fact that sensory attenuation effects were characterized by negative values, the sign of the effect was reversed and entered into the regression analysis. The independent variables were MRMFs amplitude from motor‐related regions, including precentral gyrus, postcentral gyrus, anterior and posterior cingulate cortex, inferior parietal cortex and cerebellum‐related areas. To avoid potential auditory activity in motor‐related areas, MRMFs in the active condition were subtracted from the passive condition data using the same time latency of each peak. Two factors of tolerance and the variance inflation factor (VIF) were employed to identify multicollinearity of independent variables. We confirmed that the predictors in final regression models have no collinearity based on tolerance >0.1 and VIF <10.

#### 2.8.3 DCM: Bayesian model selection

For DCM model‐analysis, fixed‐effects Bayesian model selection (FFX‐BMS) was used to determine the winning DCM‐model. The metric of model performance was the free energy approximation to the model evidence: the probability of the observed data given the model (integrating over all possible parameter values). This free energy metric is improved by model accuracy but penalized by model complexity. Each model inversion also derived the posterior distributions of the parameters given the observed data.

## 3 RESULTS

### 3.1 Sensory attenuation

**FIGURE 2:**
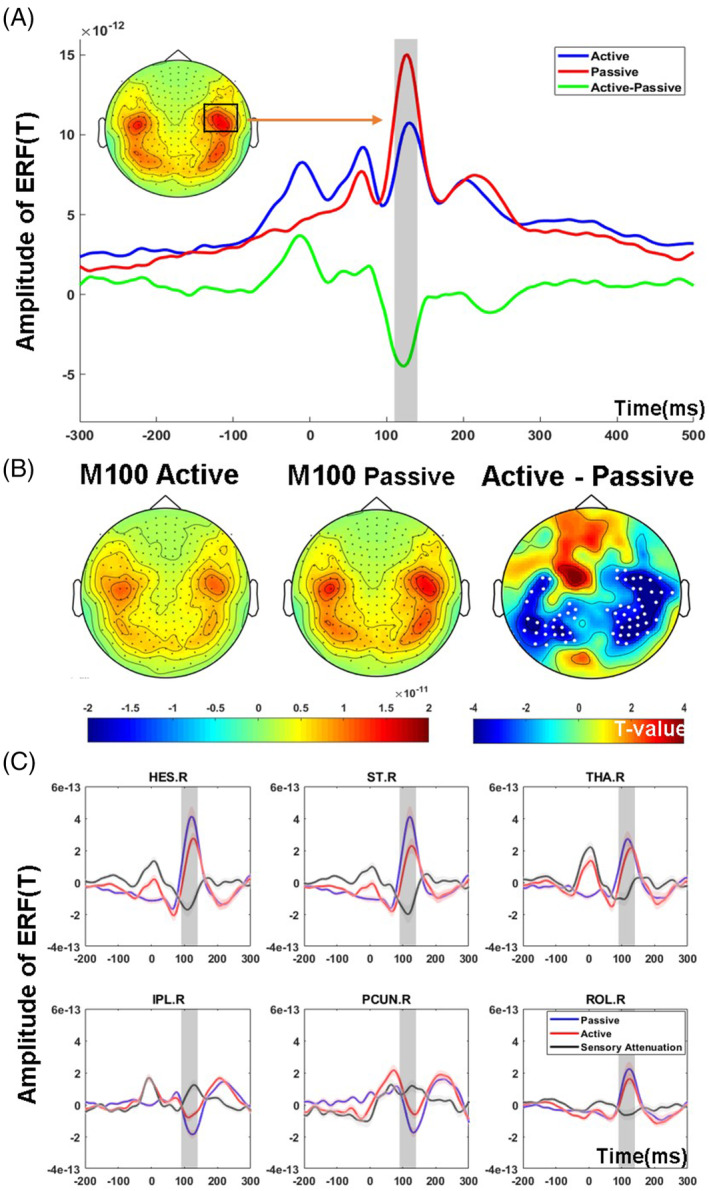
M100 sensory attenuation effects at sensor‐ and source‐space level. Panel A depicts the grand average butterfly plots of six sensors over right auditory‐parietal areas with maximal auditory activity. The gray area displays the M100 time window in the active and passive conditions between 110 and 140 ms. Panel B displays the planar topography map of the M100 in the active and passive conditions. The statistically significant channels that differ between active and passive conditions are highlighted. Panel C shows the mean and standard error of the auditory ERFs in active and passive conditions and sensory attenuation effects in parietal, auditory, and subcortical areas (after FDR correction). The gray highlighted area shows the statistical time‐window between 90 and 140 ms. Tha, thalamus; HES, Heschl's gyrus; ST, superior temporal cortex; IPL, inferior parietal cortex; Precu: precuneus; ROL, rolandic operculum; L, left; R, right

At sensor level, visual inspection of grand average ERFs revealed amplitude differences between active and passive conditions between 110 and 140 ms. This time window was then used for further analysis with a cluster‐based nonparametric permutation approach to identify channels with significant sensory attenuation. A smaller M100 amplitude was observed over temporal and parietal channels in the active condition versus passive condition (*p* < .05) (Figure 2A).

At source level, we identified significant sensory attenuation across sources between 90 and 140 ms. Monte‐Carlo nonparametric permutation results indicated that the sensory attenuation effect was present in right thalamus, right HES, right ST, right rolandic operculum (ROL) as well as in parietal regions, located in the right inferior parietal cortex (IPL) and right precuneus (Figure 2B).

### 3.2 MRMFs

**FIGURE 3:**
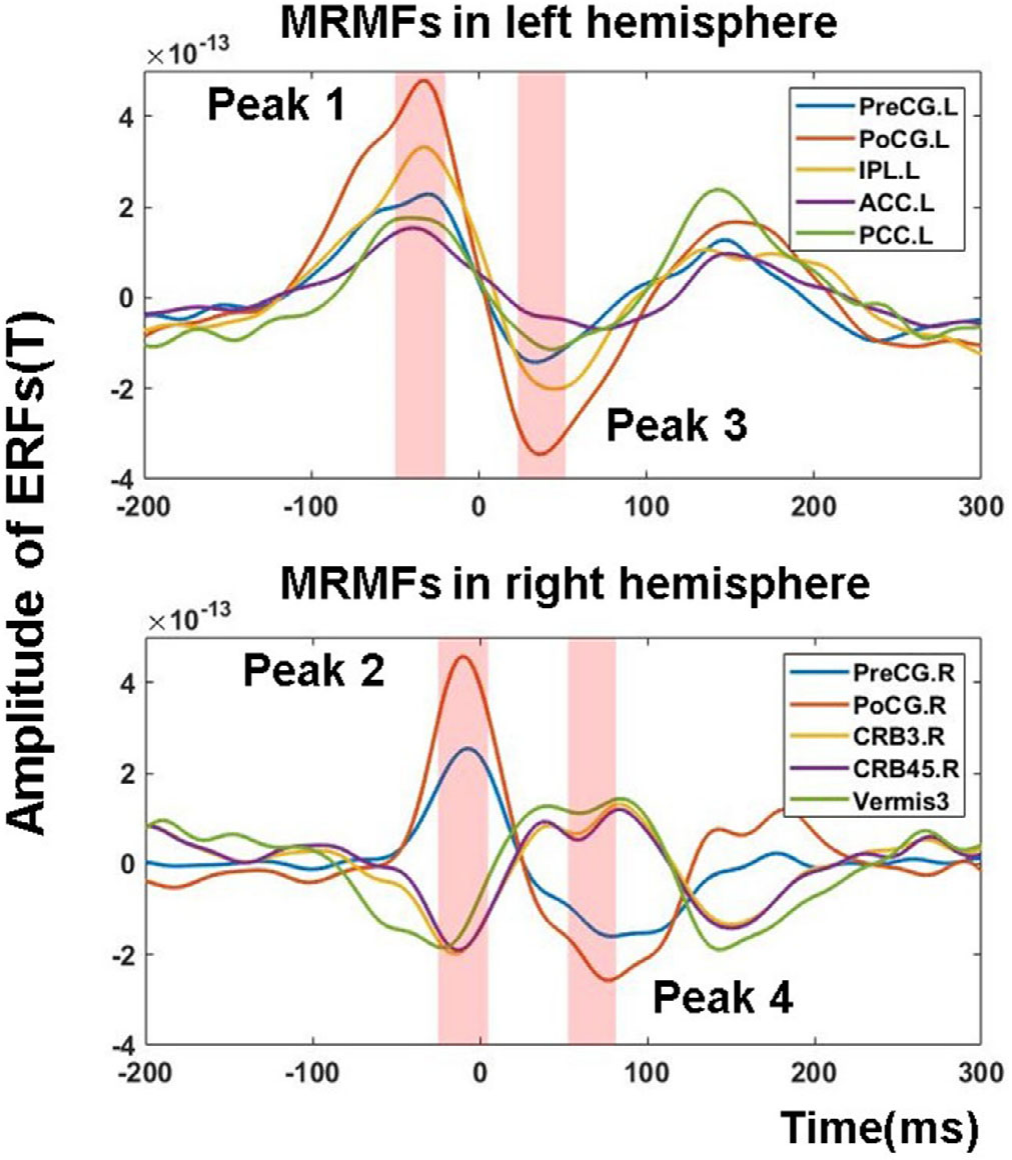
Movement‐related magnetic fields (MRMFs) peaks at source level. ROIs of MRMFs from Peak 1 to Peak 4 and the pink shadows highlights the analysis time windows (Peak 1: −50 to −20 ms; Peak 2: −25 to 5 ms; Peak 3:20 to 50 ms; Peak 4:50 to 80 ms). MRMFs: Movement‐related magnetic fields; PreCG: Precentral gyrus, PoCG: Postcentral gyrus; IPL, inferior parietal cortex; ACC, anterior cingulate cortex; PCC, posterior cingulate cortex; CRB3, lobe III of cerebellum; CRB45, lobe IV, V of cerebellum; HES, Heschl's gryus; ST, superior temporal cortex; L, left; R, right

We observed the following MRMFs: (a) Motor preparation potentials, (b) Motor potential peak, and (c) Motor re‐afference peak. The motor‐readiness potential was not included in further analysis as it could be confounded by attention, anticipation and task load (for a review see (Hughes, Desantis, & Waszak, 2013). Given the fact that we observed contralateral (left hemisphere) and ipsilateral (right hemisphere) MRMFs, we identified 4 MRMF‐related peaks, including a contralateral MRMF with a peak latency between −50 and − 20 ms (Peak 1) and a similar MRMF in the ipsilateral hemisphere with a peak latency between −25 and 5 ms (Peak 2). Additionally, the re‐afference potential in contralateral and ipsilateral hemisphere constituted Peak 3 and Peak 4 with a time latency from 20 to 50 ms and from 50 to 80 ms, respectively (Figure 3). The mean amplitude of each peak within above mentioned time window were entered into the following regression model.

### 3.3 Regression analysis

Five regions with the highest amplitude from each peak of MRMFs were selected as regions of interest (ROIs) (independent variables). As the brain regions in Peak 1 and Peak 3 as well as in Peak 2 and Peak 4 were identical, 20 MRMF‐related peaks from 10 ROIs were used in the stepwise multiple regression.

The regression model significantly predicted sensory attenuation in the right HES (adjusted *R* ^2^ = 0.39, F [2,32] =11.72, *p* < .001) and in the right ST (adjusted *R* ^2^ = 0.25, F[2,32] = 8.0, *p* = .001). Peak 2 in the right postcentral gyrus (PoCG)(Beta_**HES**_ = 0.42, *p*_**HES**_ = 0.004; Beta_**ST**_ 0.37, *p*_**ST**_ = 0.014) and Peak 3 in left precentral gyrus (PreCG) (Beta_**HES**_ = 0.47, *p*_**HES**_ = 0.001; Beta_**ST**_ = 0.40,*p*_**ST**_ = 0.008) significantly predicted sensory attenuation in the right HES and the right ST (Table 1).

**TABLE 1:** Summary of multiple regression results

	Coefficient *SE*	Std.coff beta	t	Sig	Collinearity tolerance	VIF
**Right HES sensory attenuation**						
Peak 2_PoCG.R	0.10	0.42	3.2	0.004*	0.99	1.00
Peak 3_PreCG.L	0.19	0.47	3.5	0.001*	0.99	1.00
**Right ST sensory attenuation**						
Peak 2_PoCG.R	0.12	0.37	2.6	0.014*	0.96	1.01
Peak 3_PreCG.L	0.15	0.40	2.8	0.008*	0.98	1.01

*Note:* The dependent variables are the reverse value of sensory attenuation in right HES, right ST, respectively. The independent variables are movement‐related activity at each peak. *(*p* < .05).

Abbreviations: HES, Heschl's gyrus; L, left; PoCG, postcentral gyrus; PreCG, precentral gyrus; R, right; Sig, significance; ST, superior temporal cortex; VIF, variance inflation factor.

### 3.4 DCM model structure

For the DCM‐model, we wished to implement a model as parsimonious as possible and thus concentrated on the following brain regions: (a) Bilateral thalamus, (b) Bilateral HES, and (c) right IPL. Bilateral thalamus and HES were included due to the fact that auditory stimuli were presented binaurally. Moreover, we only included HES as the ST is anatomically close to the HES and sensory attenuation in both regions was highly correlated (r = 0.88, *p* < .001). Although the attenuation of the M100 was also observed in ROL and precuneus, we did not include these regions because additional brain regions substantially increase the complexity of the DCM model, in particular if the areas distant (in hierarchical terms) from the input. Finally, as mentioned previously, brain areas displaying MRMFs, including bilateral PreCG and PoCG, left ACC, left PCC, left IPL, cerebellum‐related areas, were not included as DCM requires that the driving input for both experimental conditions is the same.

**FIGURE 4:**
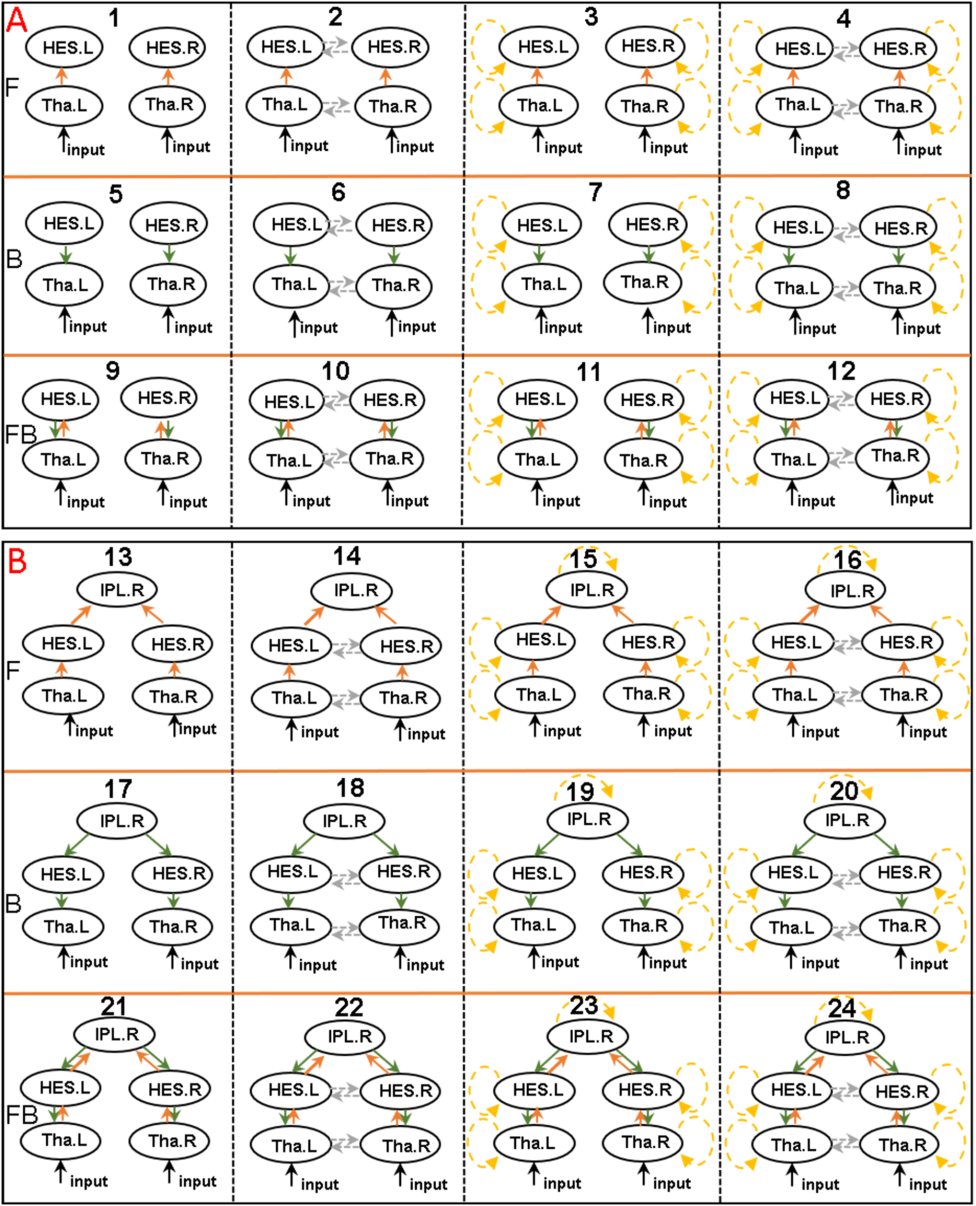
DCM‐model structures. Panel A displays the structure of family A and panel B shows the structure of family B. The rows displayed forward (F, orange solid line), backward (B, green solid line) and bi‐direction (FB) connection pattern in each family, which were then varied within or without intrinsic and lateral connection. Gray dotted line shows the lateral connection, and yellow dotted line represents self‐modulated connection. Tha, thalamus; HES, Heschl's gyrus; IPL, inferior parietal lobe; L, left; R, right. The winning model is model with number 23

DCM was then used to test the contribution of each brain area (HES, IPL, and Thalamus) toward sensory attenuation as well as the interactions between nodes to examine the role of feedback and feedforward message passing as well as the importance of intrinsic connectivity. Family A included bilateral thalamus and HES to test whether sensory attenuation was mediated by a thalamocortical network. The right IPL was then added into Family B to examine the potential role of top‐down predictions on auditory areas. In all cases, driving inputs into the bilateral thalamus conveyed the auditory stimulus which perturbs brain activity, which is then modulated by condition‐specific effects on forward, backward, or intrinsic connections. Models with or without intrinsic (self‐inhibitory) and lateral connections at each level were also included (Figure 4).

### 3.5 Fixed effect factors of BMS

**FIGURE 5:**
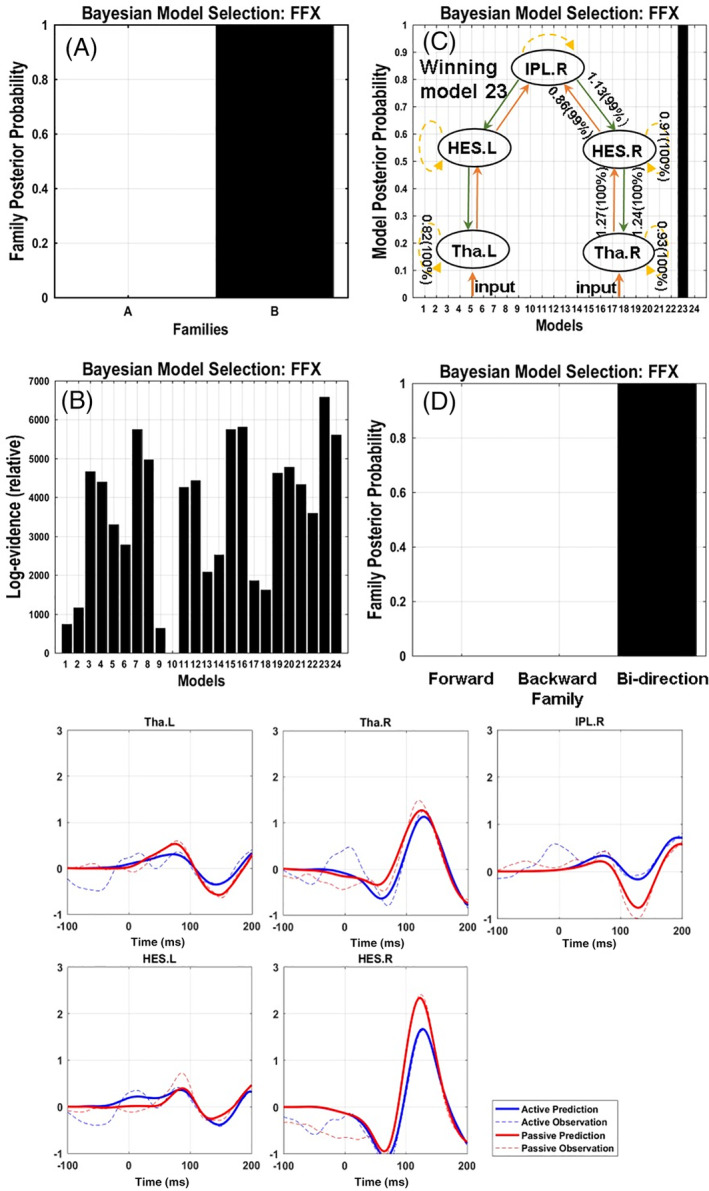
Dynamical causal modeling results: BMS results based on fixed effect (FFX) and the grand‐average ERF of predicted and observed evoked potential response in five nodes. Panel 5A displays the posterior probability at family level. Panel 5B displays the log‐evidence of individual models. Panel 5C shows the winning models across all the constructed models with almost 100% posterior probability. Simultaneously, condition inference (more than 95% posterior probability) of modulatory connection of the winning model were marked in Panel 5C (Model 23 in Figure 4). The connection parameters were described with the gain coupling and the probability that the coupling was increased (gain coupling >1) or decreased (gain coupling <1) in active condition. Panel 5D displayed the BMS results based on the forward, backward, bidirectional modulatory connection pattern in each family. Panel 5E shows the grand‐average ERFs of predicted and observed evoked potential response in five nodes. The solid and dotted line represent the predicted and observed ERF in active (red line) and passive condition (blue line). The x‐axis is the time (ms), and the y‐axis is the ERF amplitude. BMS; Bayesian model selection; Tha, thalamus; HES, Heschl's gyrus; IPL, inferior parietal lobe; L, left; R, right

At the family level, Fixed effect factors (FFX) favored Family B with nodes in IPL, thalamus and HES. At the model level, Model 23 won with almost 100% posterior probability, involving both bottom‐up and top‐down modulation connections as well as self‐modulation in each node but without lateral connections (Figure 5A–C). Additionally, we re‐organized the models into three alternative families according to the connections modulated by sensory attenuation in forward, backward and bidirectional modulation connection pattern. FFX results suggested that the family with both forward and backward modulated connections had the most evidence with 100% probability (Figure 5D). The winning model fitted the data well with the observed and predicted waveforms closely aligned in all areas (the exceptions being effects occurring prior to 0 ms (the input onset) which cannot be modeled using this approach) (Figure 5E).

Finally, the modulatory parameters were averaged across participants after Bayesian model averaging (BMA) over the winning family in order to identify the connections that were modulated in the sensory attenuation condition. Only connections with a posterior probability (of being modulated during sensory attenuation) of over 95% are reported. For the winning model, the self‐inhibition was decreased during sensory attenuation (i.e., implying increased excitability or “gain”) in bilateral thalamus and right HES, and the bottom‐up (excitatory) connection strength from right thalamus to right HES was likewise increased. Conversely, the bottom‐up connection strength from right HES to right IPL was reduced, and top‐down (inhibitory) connection strengths between right IPL, right HES and right thalamus were increased (Figure 5C).

## 4 DISCUSSION

The current study aimed to identify the brain regions and network interactions underlying sensory attenuation of the M100. Our MEG data show that sensory attenuation was present in the right HES and ST, ROL and parietal areas as well as in the thalamus. Pronounced activation of the right auditory cortex is consistent with previous data implicating the right hemisphere in the processing of simple sounds (Zatorre, Bouffard, Ahad, & Belin, 2002; Zatorre, Evans, Meyer, & Gjedde, 1992). Moreover, our analysis revealed that MRMFs involving right PoCG and the left PreCG positively predicted sensory attenuation in the right HES and ST. Finally, DCM results suggest that auditory sensory attenuation involved both top‐down and bottom‐up modulations in a thalamocortical network.

The involvement of the HES and ST is consistent with invasive electrophysiological data indicating that sensory attenuation occurs in both primary and secondary auditory cortices (Rummell, Klee, & Sigurdsson, 2016). In contrast, previous MEG studies (Aliu et al., 2009; Martikainen et al., 2004) only localized sensory attenuation to secondary auditory regions. One reason for these divergent findings may be differences in the source localization approach employed. In current study, we identified generators with a LCMV beamforming approach while previous employed an equivalent current dipole (ECD) technique.

Previous fMRI and EEG/MEG studies have observed reduced parietal cortex (Benazet, Thénault, Whittingstall, & Bernier, 2016; Blakemore, Wolpert, & Frith, 1998; Hughes & Waszak, 2011) and precuneus activity (Cao, Veniero, et al., 2017) during self‐induced sensations. The IPL is a core area for the integration of auditory‐motor information (Alain, He, & Grady, 2008; Hickok, Okada, & Serences, 2009; Pa & Hickok, 2008). Moreover, existing evidence supports that IPL plays an important role through interactions with the cerebellum (Pollok et al., 2008) in the prediction of motor outcomes (Blakemore & Sirigu, 2003). Accordingly, the involvement of IPL in the current task may index a role in the mapping of integrated auditory and motor responses.

An alternative explanation is that the IPL reflects the participants' covert analysis of time‐intervals between sounds as a strategy to respond to task demands. In either case, the observed suppression of IPL activity to self‐generated sounds may be discussed in the context of motor predictive signals, resulting in a suppression of self‐generated auditory‐motor or temporal representations. Future studies assessing involvement of efferent motor signals during auditory sensory attenuation should therefore further address the role of predictive signals in the attenuation of IPL activity.

A novel observation in our MEG‐study is the presence of sensory attenuation in the thalamus and ROL. Modulation of thalamic activity has been described during sensory attenuation in previous fMRI‐data (Blakemore, Wolpert, & Frith, 1998; Boehme, Hauser, Gerling, Heilig, & Olausson, 2019; Fu et al., 2005), but the functional role of the thalamus has remained unclear. As previously highlighted, one possibility is that the thalamus underlies the relay of the efference copy generated in motor areas to auditory regions (Sherman, 2016), which is supported by evidence from visual perception (Bellebaum, Daum, Koch, Schwarz, & Hoffmann, 2005; Sommer & Wurtz, 2004). In contrast, sensory attenuation in the ROL is likely to reflect the role of executive motor functions (Penfield & Roberts, 2014) and somatosensory processing (for a review see (Mălîia et al., 2018).

Regression analyses highlighted the contribution of the right PoCG and the left PreCG in the modulation of the M100 sensory attenuation in HES and ST. The involvement of the PoCG, a region of the somatosensory cortex, is a novel observation compared with previous evidence that have highlighted the role of motor‐related areas , including the supplementary motor cortex and premotor cortex, in sensory attenuation (Haggard & Whitford, 2004; Oestreich, Whitford, & Garrido, 2018). The contribution of the PoCG towards sensory attenuation is consistent, however, with emerging evidence that activation of somatosensory cortex is mediated by motor‐related cortex during voluntary movement (Christensen et al., 2007).

Moreover, the left PreCG also positively predicted auditory sensory attenuation. The re‐afference potential of the PreCG reflects proprioceptive afferents of motor actions (Naito, 2004) and thus could contribute to body ownership (Walsh, Moseley, Taylor, & Gandevia, 2011). Indeed, it has been proposed that body ownership mediates sensory attenuation via updating the internal body state that in turn provides input to generate sensory prediction (Kilteni & Ehrsson, 2017a). This perspective is in line with the predictive coding account that has highlighted the importance of proprioceptive afferents to guide and predict motor outcomes (Adams, Shipp, & Friston, 2013; Brown, Friston, & Bestmann, 2011).

Finally, our DCM modeling results suggest that sensory attenuation likely involves reciprocal feedforward and feedback loops between thalamus, HES and right IPL as well as intrinsic modulation within each source. Notably, Bayesian model selection identified the family model which involved interactions between the right IPL and bilateral HES. The involvement of the IPL in auditory sensory attenuation supports the view that parietal cortices provide a top‐down modulation of sensory regions (Auksztulewicz & Friston, 2015).

In terms of the extrinsic modulation of connections between sources, our DCM parameter support the enhancement of both top‐down and bottom‐up connections in the active condition, particularly in the right hemisphere. Moreover, the winning DCM model involved modulation of intrinsic (self‐inhibitory) connections, increasing local synaptic gain following the actively produced sound. Taken together, these results imply that the self‐generated stimuli entail an initial amplification of the sensory input through the thalamus that is then suppressed by an increased inhibition of this input by top‐down connections. This pattern is consistent with the source‐space data (Figure 2), where the active condition causes a greater deflection than the passive condition in the early right thalamic response (around 70 ms), which is subsequently damped, especially in higher order auditory areas at around 110 ms. Interestingly, a similar pattern was observed in an auditory oddball paradigm containing manipulations of attention and expectations (Auksztulewicz & Friston, 2015). In this study, attention had an early enhancing effect on the ERP (~50 ms), in part by changing the gain (self‐inhibition) in HES, whereas expectations had a later inhibitory effect on the ERP (~140 ms), accounted for by changes in backward (and forward) connectivity Thus, from a predictive coding account, self‐generated sensations may similarly produce an initial boost (as the precision of the predicted sensations is high) but then a subsequent dampening (as this sensory input is better predicted, reducing the prediction error).

## 5 LIMITATIONS

One potential limitation of our findings is the detection of thalamic activity with MEG. However, emerging evidence supports the ability of MEG to detect activity in deeper brain areas, such as the thalamus (Cornwell et al., 2008; Roux, Wibral, Singer, Aru, & Uhlhaas, 2013) and hippocampus (Recasens, Gross, & Uhlhaas, 2018). In addition, we did not include a motor‐only condition as a baseline for the sensorimotor‐system. However, previous studies showed that sensory attenuation remains present after ruling out the motor contamination by subtracting motor activity from motor‐auditory activity (Horváth, 2014; Martikainen et al., 2004).

We also employed a slightly wider time‐window for source‐level analyses, compared with sensor‐level data. This wider window optimally covered the M100 response across the different brain regions in the auditory processing hierarchy for which onset latency differences have been observed (Nourski et al., 2018). We would like to note, however, that the source‐level data were conservatively corrected and showed more robust sensory attenuation effects than sensor‐level estimates, most likely due to better un‐mixing of contributions from different brain regions.

In addition, the DCM‐analysis only compromised a subsection of brain regions that showed sensory attenuation effects. We intentionally selected only the HES, IPL, and thalamus since a larger number of sources would have increased the complexity of the DCM‐model significantly. Secondly, we did not include motor‐regions as indicated above as the driving input for both experimental conditions needs to be similar in DCM.

## 6 SUMMARY

Taken together, our results provide novel evidence to suggest that sensory attenuation involved a distributed network in cortical (motor, parietal, and auditory regions) as well as subcortical (thalamus) regions. Furthermore, DCM analysis revealed that self‐generated sensations are associated with information flow in a thalamocortical network that involves bottom‐up, top‐down and local self‐inhibitory connections. Specifically, the winning DCM model highlights the crucial role of the thalamus in amplifying self‐generated sensations, before this activity is attenuated (in both cortex and thalamus) by top‐down projections from auditory and parietal areas. In addition to the relevance for understanding normal brain functioning, these data provide a potential framework for the investigation of alterations in psychiatric syndromes, such as ScZ, where abnormal sensory attenuation may provide clues to the symptoms of psychosis (Ford & Mathalon, 2005).

## Data Availability

The data sets generated during and/or analyzed during the current study are available from the corresponding author on reasonable request.
